# Prevalence and characteristics of chronic body pain in China: a national study

**DOI:** 10.1186/s40064-016-2581-y

**Published:** 2016-06-30

**Authors:** Beifeng Chen, Linlin Li, Connor Donovan, Yongqing Gao, Gholam Ali, Yan Jiang, Tan Xu, Guangliang Shan, Wenjie Sun

**Affiliations:** School of Public Health, Wannan Medical College, Wuhu, 241002 China; University of California Davis Health System, Sacramento, CA 95817 USA; College of Business, University of Arkansas at Little Rock, Little Rock, AR USA; School of Food Science, Guangdong Pharmaceutical University, Zhongshan, 528458 China; School of Medicine, Tulane University, New Orleans, LA 70112 USA; Infocast Company, Kowloon, Hong Kong; Department of Epidemiology, School of Public Health and Jiangsu Key Laboratory of Preventive and Translational Medicine for Geriatric Diseases, Medical College of Soochow University, Suzhou, Jiangsu Province 215123 China; Tulane University, School of Public Health and Tropical Medicine, 1440 Canal Street, New Orleans, LA 70112 USA; Department of Epidemiology and Statistics, Institute of Basic Medical Sciences, Chinese Academy of Medical Sciences; School of Basic Medicine, Peking Union Medical College, Beijing, 100005 China

**Keywords:** Pain, Prevalence, China

## Abstract

**Background:**

Chinese citizens regularly experience some form of bodily pain, yet little is known regarding the epidemiology of pain.

**Methods:**

We conducted a nationally representative sample cross sectional study to estimate the prevalence of pain and identify risk factors of pain among 19,665 community residents aged 18–65 years in China. The China Sub-optimal Health Survey (CSHS) data was used to estimate pain prevalence. Body pain was also estimated by self-reports from the sample population. A logistical regression model was applied to estimate the odds ratio and 95 % CIs of acute pain and chronic pain to explore the potential risk factors.

**Results:**

Women had a higher prevalence of pain than men (39.92 vs. 32.17 % for chronic pain). The prevalence of pain increased with age (29.72 % for ages 18–25 vs. 42.23 % for ages 45–65). The most common complaints were head, neck/shoulder, and waist/back pain. Females (OR 1.57, 95 % CI 1.44–1.71) ages 25 or older (25–45: OR 1.19, 95 % CI 1.04–1.36; 45–65: OR 1.47, 95 % CI 1.26–1.73) were more likely to report having chronic pain. Subjects’ living areas, and their drinking status (OR 1.32, 95 % CI 1.13–1.53) or smoking status (OR 1.01, 95 % CI 0.91–1.11), were also factors that were significantly associated with increased reporting of chronic pain.

**Conclusion:**

Women had a higher prevalence of chronic pain than men, although both sexes had a high prevalence for chronic pain. There were significant differences between the two sexes and the location of chronic pain in the body, most notably in the shoulders, stomach, abdomen, and waist.

## Background

It is common for the general population to suffer from physical pain in the body. Nearly one in three adults worldwide experiences some sort of physical pain (Tsang et al. 2008). Very few studies have explored body pain in the general population, although there are some studies found in clinical settings. Previous epidemiological studies of the general population have shown that body pain varies from 8.7 to 42.0 % among adults in different countries (Yeo and Tay 2009; Cabral et al. 2014; Duenas et al. 2015). The wide variation in prevalence of pain could be partially explained by methodological, racial/ethnical, or cultural differences (Johannes et al. 2010). Evidence strongly suggests that complaints of pain are more common among the obese (Dario et al. 2015; Okifuji and Hare 2015), women (Boerner et al. 2015; Tighe et al. 2014; Oliva et al. 2015), middle aged individuals (LeResche et al. 2015; Houde et al. 2015), and individuals of lower socioeconomic status (SES) (Latza et al. 2000). However, most of the studies referenced above were from Western countries. There is limited information available on body pain in China. A community-based study conducted in Chongqing, China reported that the prevalence of body pain among the local adult population was 42.2 % (Jackson et al. 2014). A Hong Kong population-based study showed that 34.9 % of the surveyed population reported pain lasting more than 3 months (chronic pain) and an average of 1.5 pain sites in the body; 35.2 % experienced multiple pain sites, with the majority of respondents rating the legs and back as the sites with the most prevalent pain (Wong and Fielding 2011). While the results of the aforementioned studies are significant, it is difficult to draw conclusions from the results due to the lack of representativeness in the studies’ sample populations. Hence, a national population-based study was needed to examine the prevalence of chronic body pain among the Chinese population, potentially providing significant indicators that might aid in preventing this chronic health condition in Chinese citizens.

The objectives of this study were to investigate: (1) the prevalence of body pain in the general Chinese adult population, and (2) the socio-demographic, clinical, and social risk factors of body pain.

## Methods

### Study design and data collection

The methodology of the study was reported elsewhere (Sun et al. 2015). In brief, the Chinese Sub-optimal Health Study (CSHS) is a cross-sectional study, with China’s population of 1.4 billion people being represented by participants from six Chinese provinces, selected through a multi-stage, random cluster sampling design. All 31 provinces or municipalities were divided into six administrative regions (Northeast, North, East, Central South, Southwest, and Northwest) based on their geographic locations. Six provinces (Jilin, Beijing, Jiangsu, Hubei, Sichuan, and Gansu) were randomly selected from within each administrative region to represent the entire region. Within each selected province, residents, including local college students, government staff, business and farm workers, and other non-affiliated local residents, were clustered and randomly selected for the study sample. Selected participants were asked to complete a self-administered questionnaire. The data on demographic and personal characteristics were collected, including: gender, age, marital status, education, smoking status, drinking status, and health information (medical history, illnesses, and diseases that occurred during the last 12 months). Information on occupation was based on Chinese labor law.

19,665 participants were selected for the study, of which 18,631 responded and completed the questionnaire (response rate: 94.7 %). We excluded individuals who (1) were younger than 18 or older than 65 years of age; and (2) had mental illnesses that might prevent them from providing accurate information in terms of body pain. After exclusion, 16,174 eligible participants were included in the final analysis.

### Ethics statement

This study was approved by the Institutional Review Board at Peking Union Medical College and followed the tenets of the Declaration of Helsinki. Written informed consent was obtained from all participants.

### Outcome: chronic body pain

In the present study, chronic body pain was defined as ongoing and having occurred for more than 3 months (Jackson et al. 2014), which was consistent with the definition used in the literatures. Chronic pain was assessed by two questions:

1. Have you had chronic body pain? If yes, please specify the site of pain.
2. Did the body pain affect your life?

Respondents were asked to rate body pain using a Likert scale (1 = Not at all, 2 = Very few, 3 = Sometimes, 4 = Very often, and 5 = Almost all the time).

### Statistical analyses

All data analyses were performed using Windows Statistical Software Package Version 10.0 (SAS Institute, Cary, NC, USA). Participant occupation was analyzed as a categorical variable. Body pain was re-categorized into two groups (*Yes/No*), which were investigated as binary outcome variables. Health status was assessed based on self-reported chronic conditions including: hypertension, diabetes, coronary heart disease, hyperlipidemia, hepatitis, and other diseases. Participants with any of the chronic diseases mentioned above were classified as being in an “unhealthy” condition.

Chi square tests were used to compare the demographic characteristics between participants who have acute body pain and those who have chronic body pain. Tukey’s tests were used to compare the difference between sub-groups. Logistical regression models were applied to examine the potential risk factors (sex, age, occupation/education, area, marriage, smoking status, drinking status, and health status) of chronic body pain. All risk factors were categorized as in Table 1 (A significance level of 0.05 is required to allow the variable to enter into the model). All the tests were two sided and the significance level was set at 0.05.

**Table 1:** Prevalence of chronic pain and percentage of affected individuals

	Prevalence	Affected
Sex		
Men	32.17	29.21
Women	39.92	34.72
Age (years)		
18–25	29.72	33.15
25–≤45	36.97	30.85
45–≤65	43.23	33.63
Occupation		
Civil	37.88	30.42
Profession	41.35	33.69
Worker	37.75	31.48
Famer	33.92	32.32
Business man/service	31.15	27.65
Students	30.84	35.79
Others	33.70	30.37
Education		
Liberate/primary school	35.80	31.27
High school	34.69	30.61
College	36.74	32.87
Area		
Jilin	56.15	37.21
Gansu	33.15	32.90
Sichuan	32.36	33.32
Jiangsu	21.32	22.86
Hubei	39.41	34.98
Beijing	40.37	31.42
Marriage		
Single	30.84	33.23
Married	38.38	31.09
Devoice/separate/Widow	43.73	37.10
Smoking		
No	36.52	32.79
Yes	34.44	29.27
Drinking		
No	35.99	32.79
Yes	36.11	30.09
Healthy		
Yes	32.24	29.55
No	48.78	40.05

## Results

The study included a total of 16,174 Chinese adults, 50.3 % male and 49.7 % female, ages 18–65 with a mean age of 33.2 (SD = 10.5). Table 1 shows the prevalence of chronic body pain by each characteristic. Women had a higher prevalence of chronic pain than men (39.9 vs. 32.2 %). The prevalence of pain increased with older age (29.72 % for ages 18–25 vs. 42.23 % for ages 45–65). Professional workers (41.4 %) had the highest prevalence of chronic pain compared to other occupations, while students had the lowest prevalence (30.8 %). Smokers, drinkers, and people in “unhealthy” conditions also had a higher prevalence of chronic pain compared to their counterparts.

Results of univariate analyses showed that only one of the seven socio-demographic variables (religion) did not meet the pre-selection criteria (P < 0.05). Hence, all the other six socio-demographic variables were entered in the multivariate model (Table 2). Females (vs. males) (OR 1.57, 95 % CI 1.44–1.71), and individuals aged 25 or older (vs. ages 18–24) (25–45: OR 1.19, 95 % CI 1.04–1.36; 45–65: OR 1.47, 95 % CI 1.26–1.73) were more likely to report having chronic pain.

**Table 2:** Factors associated with pain

	Chronic pain
	Adjusted OR
Sex	
Men	Ref
Women	1.57 (1.44–1.71)*
Age (years)	
18–25	Ref
25–≤45	1.19 (1.04–1.36)*
45–≤65	1.47 (1.26–1.73)*
Occupation	
Civil	0.87 (0.77–0.98)*
Profession	1.13 (1.00–1.28)*
Worker	Ref
Famer	0.98 (0.85–1.14)
Business man/service	0.95 (0.82–1.09)
Students	1.03 (0.88–1.20)
Others	0.90 (0.78–1.04)
Education	Ref
Liberate/primary school	1.01 (0.91–1.12)
High school	1.08 (0.96–1.20)
College	
Area	
Jilin	Ref
Gansu	0.44 (0.39–0.49)*
Sichuan	0.36 (0.32–0.41)*
Jiangsu	0.25 (0.22–0.28)*
Hubei	0.57 (0.51–0.64)*
Beijing	0.56 (0.49–0.63)*
Marriage	
Single	0.94 (0.83–1.06)
Married	Ref
Devoice/separate/widow	1.11 (0.90–1.37)
Smoking	
No	Ref
Yes	1.01 (0.91–1.11)
Drinking	
No	Ref
Yes	1.10 (1.00–1.20)*
Healthy	
Yes	Ref
No	1.94 (1.78–2.11)*

Respondents with chronic diseases were more likely to report acute pain compared to those without chronic diseases (OR 1.94, 95 % CI 1.78–2.11). Drinking alcohol (OR 1.32, 95 % CI 1.13–1.53) and smoking cigarettes (OR 1.01, 95 % CI 0.91–1.11) were factors significantly associated with increased odds of chronic body pain.

The sites of the body pain are shown in Table 3. For men, the main sites for body pain were in the head (29.7 %), neck and shoulders (24.5 %), followed by waist and back (22.5 %), and then the stomach (17.2 %). For women, the main sites for body pain were in the head (36.7 %), neck and shoulders (32.4 %), followed by waist and back (26.4 %), and then the stomach (20.7 %).

**Table 3:** The percentage of pain in various body locations in the two sexes

	Male	Female	Total	P value
Pain site				
Head	29.65	36.71	33.13	<0.0001
Shoulder	24.49	32.41	28.39	<0.0001
Stomach	17.23	20.72	18.95	<0.0001
Abdominal	9.06	16.86	12.90	<0.0001
Waist	22.52	26.38	24.42	<0.0001
Joint	14.66	14.58	14.62	0.12
Limbs	12.69	12.23	12.46	0.08
Chest	6.88	6.19	6.54	0.02
Others	0.39	0.43	0.41	0.64

* P for the difference between sexes

## Discussion

Our results suggest that 35.9 % of the Chinese population suffers from chronic body pain and the pain is mostly prevalent around the head, neck/shoulders, and waist/back. Individuals who are female or older, who have a low SES, or who have chronic diseases are independently associated with a higher prevalence of body pain; the overall prevalence of chronic body pain in our study is very close to previous findings on chronic body pain found in Chinese adults in certain areas, such as in Hong Kong (from 34.9 to 45.9 %) (Wong and Fielding 2011; Chung and Wong 2007), Taiwan (42.0 %) (Yu et al. 2006), and Chongqing (25.8 %) (Jackson et al. 2014). Of note, a Singapore study reported that the prevalence of pain among.

Chinese in Singapore was 8.7 % (Yeo and Tay 2009), which was much lower than the prevalence in other areas. However, this study excluded people who were living in nursing homes and who were cognitively impaired, which could potentially under-estimate the prevalence of chronic body pain. Breivik et al. (2006) conducted a large-scale computer-assisted telephone survey to explore the prevalence, severity, treatment and impact of chronic pain in 15 European countries and Israel. They found that 19 % of 46,394 respondents had suffered pain for 6 months. Compared to the countries in Breivik et al.’s study, the prevalence of chronic pain in China is high. However, Breivik et al. reported that 61 % of respondents were less able or unable to work outside the home, which is higher than the result found in this study. The different results from these studies could be attributed to differences in sample populations, sampling methods, measurements, and culture factors.

Our results also indicated that the main sites for body pain were in the head, neck/shoulders, and waist/back. Jackson et al. (2014) found that the most common complaints among Chinese people in Chongqing came from back pains (17.6 %), headaches (14.2 %), joint pains (10.5 %), and abdominal pains (10.4 %). Additionally, in our sample, 8.4 % of people reported that acute pain affected their lives, and 31.9 % of people reported that chronic pain affected their lives. The combined prevalence of body pain is consistent with previously reported findings. For example, a Spanish study found that 47.2 % of Spanish people indicated that body pain was affecting their family life (Duenas et al. 2015). However, this data was not comparable to our results due to inconsistent sampling methodology and different study population.

Our study also described potential risk factors that might lead to a greater prevalence of pain, e.g. being female, aging, and being deemed “unhealthy,” which were also reported by previous studies (Yeo and Tay 2009; Cabral et al. 2014). In our sample, compared to workers, the prevalence of body pain among civil servants was significantly lower. A few previous studies reported similar findings indicating that complaints of body pain could differ by occupation (Yue et al. 2012; Liu et al. 2012). Our results also suggested that low SES was associated with higher rates of chronic pain, which was consistent with previous studies (Bonathan et al. 2013). Of note, a person’s occupation (one important component of socioeconomic status) is associated with lower back pain. For instance, Jin et al. (2004) conducted a cross-sectional study in China and found that garment workers had a higher annual prevalence of lower back pain (74 %) than teachers (40 %) (PR 1.9, 95 % CI 1.4–2.4); this could be explained by the garment workers’ long duration of sedentary positions. Miljković et al. (2014) investigated the association of experimentally induced pain threshold and tolerance with socioeconomic status, and their results suggested that poor people have a higher prevalence of pain. A significant association was found between the experimentally induced pain threshold and tolerance and marital socioeconomic status. This finding indicated that poor people indeed do hurt more. Our study could have major public health implications (Riskowski 2014). Due to the aging of the Chinese population, body pain will increase significantly in the coming future. Chen et al. (2015) conducted a study on physical therapy and lower back pain among patients in Guangdong, China. They found that the willingness-to-pay for acupuncture and low-frequency infrared treatment was about $618.6 and $592.4 USD per course, respectively. It demonstrated patients’ demand for pain management. Lacking effective pain management, the recurrence of the body pain is high (Chu et al. 2015). Further studies on pain management are warranted.

The strengths of this study include the large sample size and the adjustments for other important confounders (age, sex, occupations, etc.). Theoretically, chronic body pain should be measured by intensity. However, in the practice, there are no standardized objective criteria to measure the intensity of chronic pain due to the inconsistency of individuals’ sensitivity to pain; this was addressed in this study. In addition, this study also measured the frequency of chronic pain in individuals, an aspect of body pain that needed further evaluation.

This study does have several limitations. Pain data was self-reported which can be imprecise and subject to reporting bias. Furthermore, there are no validated tools for assessing self-reported pain. Future studies with pain scales are warranted. In addition, data on depression (Chen et al. 2012) and pain killers, which could be potential confounders, was not available. Lastly, adverse life events and psychological distress could have had mediating effects on the association between individuals’ socioeconomic status and their reported body pain (Macfarlane et al. 2009).

## Conclusion

The prevalence of body pain among Chinese adults was high. Being female, aging, having a low SES, and possessing chronic diseases were independent factors associated with higher prevalence of pain. Women had a higher prevalence of chronic pain than men, although both sexes had a high prevalence for chronic pain. There were significant differences between the two sexes and the location of chronic pain in the body, most notably in the shoulders, stomach, abdomen, and waist.
